# Magnetically Driven Soft Continuum Microrobot for Intravascular Operations in Microscale

**DOI:** 10.34133/2022/9850832

**Published:** 2022-02-15

**Authors:** Dan Liu, Xiaoming Liu, Zhuo Chen, Zhaofeng Zuo, Xiaoqing Tang, Qiang Huang, Tatsuo Arai

**Affiliations:** ^1^Key Laboratory of Biomimetic Robots and Systems, Ministry of Education, State Key Laboratory of Intelligent Control and Decision of Complex System, Beijing Advanced Innovation Center for Intelligent Robots and Systems, and School of Mechatronical Engineering, Beijing Institute of Technology, Beijing 100081, China; ^2^Center for Neuroscience and Biomedical Engineering, The University of Electro-Communications, Tokyo 182-8585, Japan

## Abstract

Remotely controlled soft continuum robots with active steering capability have broad prospects in medical applications. However, conventional continuum robots have the miniaturization challenge. This paper presents a microscale soft continuum microrobot with steering and locomotion capabilities based on magnetic field actuation. The magnetically driven soft continuum microrobot is made of NdFeB particles and polydimethylsiloxane (PDMS), and it can be as small as 200 *μ*m in diameter. Moreover, a hydrogel layer is covered on the surface of the microrobot, which not only overcomes the adhesion force between the microobjects and the soft tip but also reduces the friction between the microrobot and substrate. The performance test indicates the soft continuum microrobot featured excellent control and steering capabilities. The experimental results demonstrate that the soft continuum microrobot can travel through the microfluidic channel by its own vibration and flexibly steer in a bifurcation environment. Moreover, the micromanipulation of microbeads in the microfluidic channels proves that the proposed microscale soft continuum microrobot has a great potential for intravascular manipulation.

## 1. Introduction

**Figure 1:**
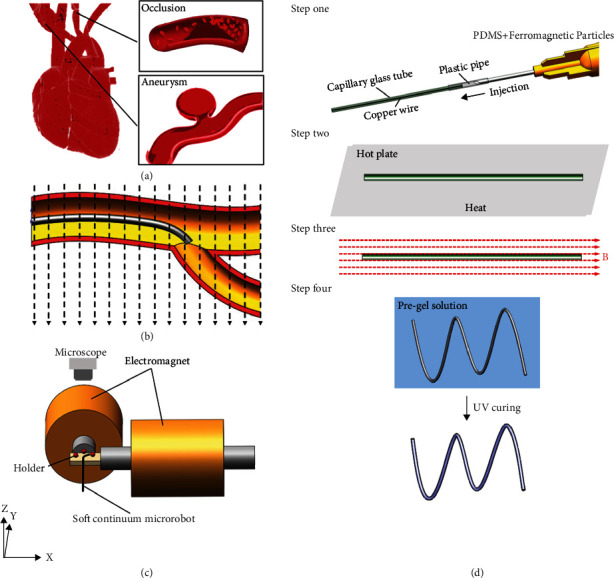
The schematic diagram of the magnetically driven soft continuum robot. (a) The intravascular disease in hard-to-reach areas where the soft continuum microrobot can be utilized. (b) The schematic of the steering of the soft magnetic microrobot in the cardiovascular system. (c) The schematic of the magnetic manipulation system. (d) Fabrication methods based on injection molding. Step one: injection molding; step two: heating for solidification; step three: magnetization; step four: forming the hydrogel skin.

In recent years, cardiovascular diseases (Figure 1(a)) have been increasing worldwide. As a result, vascular therapy has become a prevalent medical method [1, 2]. In vascular therapy, the surgeon needs to manually guide a guidewire less than one millimeter into the blood clot. However, intravascular surgery has strict requirements on the size and motion control of the device [3–5]. Therefore, microscale soft continuum manipulators hold promise for cardiovascular diseases due to being minimally invasive and safe.

In general, continuum manipulator actuation includes tendon driven [6], hydraulic driven [7], pneumatic driven [8], shape memory [9], and magnetic field actuation [10]. Camarillo et al. [11] researched a mechanics modeling of a tendon-driven continuum-robotic manipulator, and the mechanics modeling was used to control a single section manipulator. Ikuta et al. [12] utilized the pressure pulse to drive the hydraulic catheter. Chen et al. [8] designed a pneumatic driven robotic manipulator for semiautonomous colonoscopy. Crews and Buckner [13] proposed a shape memory alloy method to actuate robotic catheters. However, for these actuated systems, each additional degree of freedom of the manipulator requires an additional driveline, which causes challenges in flexibility and miniaturization of the manipulator. In recent years, magnetic field actuation gains widespread popularity in continuum manipulator control because of its wireless actuation and large driving force.

Initially, manually controlled magnetic fields were used to manipulate a magnetically tipped catheter in the vasculature [14–16]. Later, magnetic resonance imaging (MRI) was used to control catheter guidance remotely [17–19]. Currently, numerous researches [20–22] have proven that the magnetic field can well overcome the miniaturization challenge caused by the large actuation mechanism. On the other hand, magnetic field actuation allows the manipulator to be composed of soft materials [23–25], which prevents undesired trauma when manipulating in soft tissue. Kratchman et al. proposed a magnet-tipped actuated elastic rod that can achieve complex three-dimension trajectories and avoid obstacles [26]. Choi et al. developed a tethered magnetically actuated microrobot that was driven by an external magnetic field for the steerability of the intravascular guidewire [27, 28]. Kim et al. proposed a submillimeter scale soft tethered robot that has the capacities of active steering and navigation [29]. The existing soft continuum microrobots were in submillimeter size and can remove blood clots in the blood vessels. However, it is hard to remove small blood clots in medicine. In order to address the issues of the soft continuum robots at the microscale, it is necessary to develop a submicron-scale microrobot to manipulate smaller objects in complex environments.

In this paper, we developed a soft magnetic microrobot based on magnetic field actuation. The proposed system can not only control the forward movement and steering of the soft continuum microrobot in complex environments but also enable the microrobot to operate microobjects. The performance of the soft continuum microrobot is measured by simulation and experiment. The traveling experiment proves the high flexibility of the proposed soft continuum microrobot in the bifurcation environment. Manipulating the microbeads with the soft continuum microrobot in the workspace illustrates that the proposed method has great potential for medical application.

This paper is organized as follows: Section 2 describes the theory of the magnetic field drive.  Section 3 introduces the fabrication process and the characteristic analysis. The experiments and discussion are given in Section 4. And conclusions are presented in Section 5.

## 2. Magnetically Driven Soft Continuum Microrobot

As shown in Figure 1(c), the magnetically driven system consists of a pair of vertical electromagnets. The soft continuum microrobot steers and moves in response to the magnetic field. The bending capacity of the soft continuum microrobot is determined by the stiffness of the microrobot, which enables it to be flexibly steered in a complex bifurcation environment.

When applying a magnetic field, the magnetic force *F*_*m*_ and the magnetic moment *M* on the soft microrobot are given by

(1) $$\begin{array}{c} F_{m}=\left(M\cdot \nabla \right)B, \end{array}$$

(2) $$\begin{array}{c} T_{m}=M\times B, \end{array}$$

where *B* is the magnetic flux density and *M* is the magnetic moment of the soft microrobot. From Equations (1) and (2), we can know that the magnetic force is proportional to the magnetic field gradient, and the magnetic torque is related to the magnetic field. The magnetic moment induces magnetic stress *σ*^magnetic^. The magnetic stress deforms the soft continuum microrobot, and it can be estimated as

(3) $$\begin{array}{c} \sigma^{\text{magnetic}}=-B⊗\Gamma M, \end{array}$$

where ⊗ means the dyadic product and Γ is the deformation gradient tensor. On the other hand, the deformation of the soft continuum microrobot generates the elastic stress *σ*^elastic^, and the elastic stress is given by

(4) $$\begin{array}{c} \sigma^{\text{elastic}}=G{\Gamma \Gamma }^{T}-pl, \end{array}$$

where *G* is the shear modulus, Γ^*T*^ is the transpose of Γ, *l* is the identity tensor, and *p* is the hydrostatic pressure.

Ignoring the inertia effects, the total stress can be expressed as

(5) $$\begin{array}{c} \sigma =\sigma^{\text{magnetic}}+\sigma^{\text{elastic}}. \end{array}$$

## 3. Fabrication and Characteristic Analysis

This part introduced the fabrication procedure, and the characteristics of the soft microrobot are evaluated.

### 3.1. Fabrication Process

The materials of the proposed soft continuum microrobot contained polydimethylsiloxane (PDMS) and neodymium-iron-boron (NdFeB) particles (Ø 5 nm). Figure 1(d) shows the fabrication process. *Step 1*: the unconsolidated PDMS and unmagnetized NdFeB particles were mixed. The stiffness and bending capacity of soft continuum microrobot are determined by the concentration of PDMS and NdFeB particles. First, the concentration of NdFeB needs to be determined, which affects the magnetization strength and flexibility of the microrobot. Then, the ratio of PDMS and the curing agent is adjusted to achieve the desired stiffness. Finally, the weight ratio of the PDMS and unmagnetized NdFeB particles was 1 : 1, and the weight ratio of PDMS and curing agent was 10 : 1. The microrobot could be manufactured by injection molding, which requires extruding the mixture through a syringe by applying pressure. In the fabrication process, a glass capillary with an inner diameter of 200 *μ*m was used as a mold, into which we injected the mixture. *Step 2*: the mold was placed on a hot plate with a temperature of 200°C for two minutes. *Step 3*: after the mixture solution had solidified, the mold with solidified material was placed under a strong magnetic field. The purpose of the magnetization is to align the magnetic moment of the solidified material with the direction of the applied magnetic field. The applied magnetic field strength was 1.5 T. We removed the glass mold after magnetization to obtain the soft continuum microrobot. *Step 4*: the microrobot was immersed in a hydrogel solution and exposed to ultraviolet radiation. Finally, a thin film of hydrogel was generated on the surface of the soft continuum microrobot. The coated hydrogel layer could not only reduce the surface friction and ensure biocompatibility but also overcome the adhesion between the tip of the continuum microrobot and the microbeads. Therefore, the proposed soft continuum microrobot can be controlled to navigate through the narrow vasculature based on magnetic actuation as shown in Figure 1(b).

### 3.2. The Simulation of the Magnetic Field and the Microrobot Deformation

The magnetically driven soft continuum microrobot deformed following the direction of the magnetic field. Therefore, the magnetic field direction is also a factor that affects the bending of the microrobot. To analyze the magnetic field generated by the two vertical electromagnets and the deformation of the soft continuum microrobot before equipment construction, the magnetic field module and solid mechanics were implemented to analyze the magnetic field direction of the workspace and deformation of the microrobot in Comsol Multiphysics. According to the magnetic properties and the structure of the electromagnet, a simple 3D model was built. The copper coil turns and input current are 1000 and 5 A, respectively, and the diameter of the soft continuum microrobot was 200 *μ*m.

**Figure 2:**
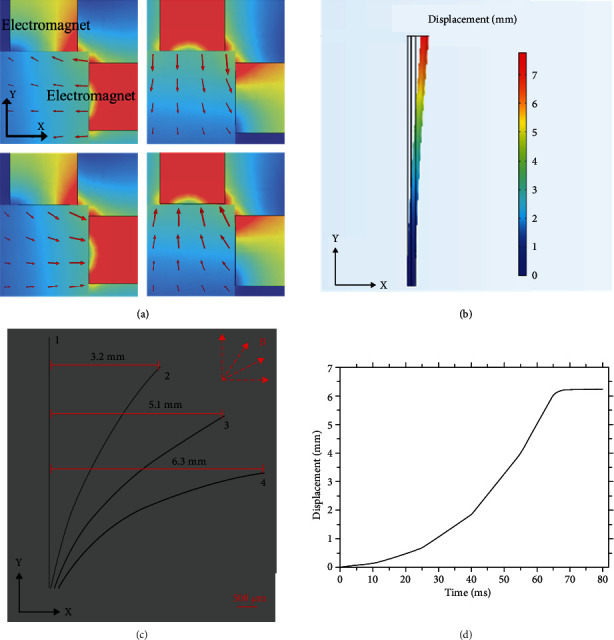
Schematic diagram of the magnetic response tip of a soft microrobot. (a) Simulation of the magnetic field along the negative *x*-axis, negative *y*-axis, positive *x*-axis, and positive *y*-axis. (b) Simulation of the continuum microrobot bending. (c) The bending of the actual soft tip under the applied magnetic field. (d) The displacement response to the current of 4A.

As shown in Figure 2(a), the red electromagnet means the electromagnet was energized, and the red arrow indicates the magnetic field direction. The simulation results demonstrate that the electromagnet system can generate a magnetic field in four directions in the plane.

To analyze the deformation of the soft continuum microrobot, the magnetic stress and the elastic stress as user-defined elements are implemented in Comsol. The simulation result of the end displacement resulting from the magnetic stress and the elastic stress is shown in Figure 2(b). It is clearly shown that the displacement of the soft continuum microrobot end was 7 mm, which demonstrates that our microrobot has a significant bending capability.

### 3.3. The Deformation of the Microrobot

The soft continuum microrobot tip was magnetized along its axis in a 1.5 T magnetic field. Therefore, the microrobot would deflect in the field direction when an external magnetic field was applied.

To verify the hypothesis of the deformation, the soft continuum microrobot was fixed to a rigid holder, and the deformation of the microrobot was observed under the digital camera. As shown in Figure 2(c), the external magnetic field caused a deformation of the soft microrobot. The deformation angle was determined by the magnetic field direction and magnetic field strength. The bending degree of the microrobot increased nonlinearly when the magnetic field direction varied from 0° to 90°. Affected by the microrobot's elastic modulus, the greater the deformation, the larger the elastic resistance. Ten tests were carried out in different magnetic field directions (±30°, ±60°, ±90°), and the bending resolution and accuracy were 3° and 2.2°, respectively. Figure 2(d) showed the step response of the microrobot tip when a current of 4 A was applied to the electromagnet. The curve demonstrated that the microrobot reached a steady state within 70 ms, which shows excellent dynamic characterization. Compared with the simulated bending, the experimental bending deflection is basically consistent with it.

The deformation experiment confirmed that the soft continuum microrobot could be steered flexibly under an external magnetic field.

## 4. Experiments and Discussion

In this part, the key functions of the soft magnetic microrobot were demonstrated, including steering and locomotion in complex environments and manipulating microobjects.

### 4.1. System Setup

**Figure 3:**
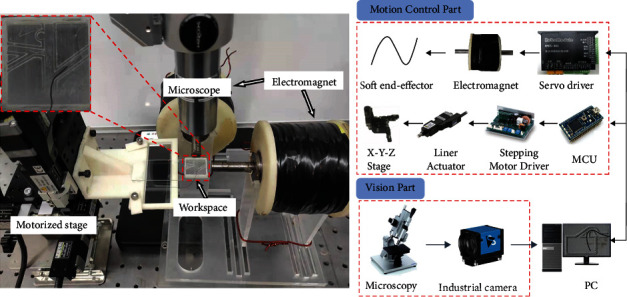
Experimental system setup.

As depicted in Figure 3, the system in this paper mainly includes two parts: the motion control part and the vision part. The motion control part consists of a pair of homemade electromagnets for producing the magnetic field. The turns of the electromagnet are 1000, and the diameter of the copper wire is 1.6 mm. The servomotor driver (RMDS-402, RoboModule) is employed to control the input current on the electromagnet, and a 12 V switching power supply (LRS-350-24, Mean Well) provides the power to the servomotor driver. Bending and vibration of the microrobot are caused by a constant and time-varying current generated by the servomotor driver, respectively. The position of the microrobot is actuated by an X-Y-Z motorized stage (TAM-655, Sigmakoki, Japan). The X-Y-Z motorized stage is driven by three stepping motors (PKP523N12B, Oriental Motor, Japan), and the stepping motor is powered by a stepping motor driver (SG-55MA, Sigmakoki, Japan) that is controlled by an MCU (mbed NXP LPC1768). The servo driver and the MCU receive the commands from the PC.

The vision part consists of monocular microscopy (CX-10C, Hirox) with a 140x objective lens and a digital camera (DFK 23U274, Imaging Source). The vision part was utilized to record the experimental data.

### 4.2. Steering and Locomotion in a Microfluidic Channel

In order to test the steering and locomotion of the soft continuum microrobot in a bifurcation environment, a microfluidic channel with the width of 800 *μ*m was fabricated by 3D printing process. The experiments were carried out in sodium dodecyl sulfate (SDS) solution. In the whole process, magnetic fields with different directions were used to control the steering of the soft continuum microrobot. The forward locomotion of the microrobot was achieved by vibrating the microrobot end and simultaneously pushing the proximal end. A time-varying magnetic field was employed to vibrate the microrobot, which eliminates the adhesion force between the microrobot and the environment. The frequency of the oscillating magnetic field was set to 10 Hz, and the amplitude of the microrobot at the frequency was about 100 *μ*m.

**Figure 4:**
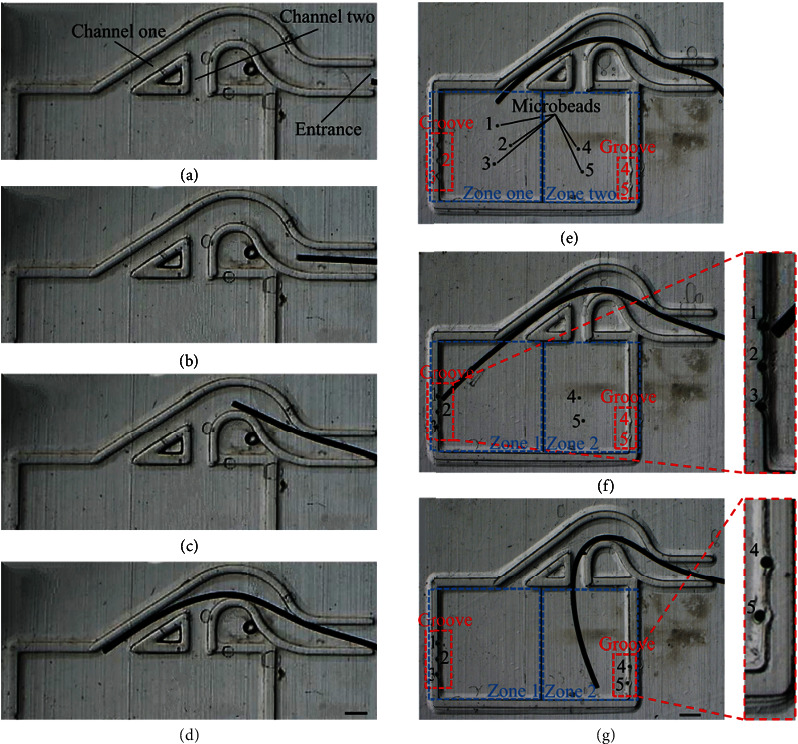
Experiments through a microfluidic channel and manipulating microbeads. (a) The soft continuum microrobot arrived at the entrance. (b–d) The process of travel based on magnetic steering capability. (d) The soft continuum microrobot entered the workspace. (e) Initialization: the soft continuum microrobot entered the workspace through a microfluidic channel. (f) Three microbeads were pushed into the desired location. (g) Five microbeads were pushed into the desired location. The scale bar represents 500 *μ*m.

As shown in Figure 4(a), the soft continuum microrobot driven by the magnetic field slowly approached the entrance of the channel. Figure 4(b) showed the locomotion of the soft continuum microrobot, and the microrobot moved forward by vibration in the channel. The microrobot encountered a bifurcation as shown in Figure 4(c). A magnetic field parallel to channel 1 was generated by controlling the two electromagnets and the tip of the microrobot pointed to channel 1. Figure 4(d) demonstrates the soft continuum microrobot moving along channel 1 after being steered. Finally, the soft continuum microrobot entered one channel of the multiple bifurcations according to the instruction and moved to the microfluidic channel exit. In conclusion, the soft continuum microrobot exhibited large bending characteristics and can steer and have locomotion in a complex bifurcation environment.

### 4.3. Manipulating Microbeads with the Soft Continuum Microrobot

To verify the manipulate capacity of the soft continuum microrobot, the polystyrene (PS) microbeads with 100 *μ*m diameter were employed. In the experiment, the same experimental setting as the steering was utilized. There are five semicircular grooves in the workspace, and the diameter of the groove is approximately 200 *μ*m. We control the direction and oscillation period of the magnetic field to push the microbeads to the destination of the microstructure.

As shown in Figure 4(e), the soft continuum microrobot crossed the microfluidic channel into the workspace. There were five grooves with the diameter of 300 *μ*m in the workspace, and five microbeads were randomly placed. As shown in Figure 4(f), the soft continuum microrobot entered zone one of the workspace through channel 1 and pushed the microbeads labeled 1, 2, and 3 into the corresponding groove. Similarly, the soft continuum microrobot traveled through channel 2 into zone 2 and pushed the microbeads labeled 4 and 5 into the groove as shown in Figure 4(g). The entire operation process was accomplished in about 10 minutes, and the average operation time of each microbead was 2 minutes.

### 4.4. Discussion

This paper proposed a submicron-scale soft continuum microrobot with steering and locomotion capabilities based on magnetic actuation. The experimental results showed that the proposed magnetically driven micromanipulation system could be used for the manipulation of microobjects. The soft continuum microrobot with flexible bending characteristics and high dynamic response has broad application prospects in the clinic treatment of intravascular diseases.

Different from the untethered microdevices, the proposed tethered soft microrobot not only interacts with the environment and undergoes large deformation in a continuum way but also possesses robust motion and reliability [9, 10]. Moreover, magnetic field actuation avoids the small driving force and execution of electrical actuation, graphene actuator, and thermal bimorph actuator [12–14]. Compared with the other tethered microdevices actuated by pneumatic driven [8], tendon driven [11], and shape memory [13], the proposed method is also evident. The advantages of these tethered microdevices are controllability of joints in flexion and extension, while the disadvantages are redundant actuation [6, 11], large size [7, 12], complex system setup [8], or hysteretic dependencies between strain, stress, and temperature [9, 13]. Our method can control the microrobot flexibly and features large driving force and miniaturization. The other tethered microdevices that were driven by magnetic field are still limited to large size [26–29].

However, there are still some restrictions. In order to operate smaller microobjects, the size of the soft microrobot is required to be smaller. Besides, the control precision of the soft continuum microrobot needs to be improved.

## 5. Conclusions

In this paper, the proposed magnetically driven soft microrobot can perform micromanipulation based on the magnetic field actuation. The soft microrobot can not only travel in complex bifurcation environments but also operate the microobjects. The steering of the soft continuum microrobot is controlled by the magnetic fields, and the locomotion of the microrobot is driven by vibrating itself. The coated hydrogel layer reduces the surface friction and ensures biocompatibility. Experiments including steering, locomotion, and manipulation of the microbeads in the microfluidic channel demonstrate that the proposed method has potential application value in intravascular disease. We envisage that the proposed soft continuum microrobot has great potential in medical applications.

## Data Availability

The data used to support the findings of this study are included within the article.
